# Supplementary material for: CRISPR/Cas13a-based supersensitive circulating tumor DNA assay for detecting EGFR mutations in plasma
Source: Commun Biol. 2024 May 28;7:657. doi: 10.1038/s42003-024-06368-2 (PMC11133305; doi:10.1038/s42003-024-06368-2)
Supplement: Supplementary file 2 — Supplementary Information [file 42003_2024_6368_MOESM2_ESM.pdf]

Supplementary Information for

CRISPR/Cas13a-based supersensitive circulating tumor DNA assay for detecting *EGFR* mutations in plasma

Li Wang[1]†, Xiaosha Wen[2,3]†, Yang Yang[2,3], Zheng Hu[4], Jing Jiang[4], Lili Duan[4], Xiaofen Liao[4], Yan He[2,3], Yaru Liu[2,3], Jing Wang[2,3], Zhikun Liang[5], Xiaoya Zhu[5], Quan Liu[2,3*], Tiancai Liu[1*], Dixian Luo[2,3*]

[1]Key Laboratory of Antibody Engineering of Guangdong Higher Education Institutes, School of Laboratory Medicine and Biotechnology, Southern Medical University, Guangzhou, Guangdong, P. R. China.

[2]Department of Laboratory Medicine, Huazhong University of Science and Technology Union Shenzhen Hospital (Nanshan Hospital), Shenzhen 518052, P. R. China.

[3]Shenzhen University Medical School, Shenzhen 518060, P. R. China.

[4]Translational Medicine Institute, the First People's Hospital of Chenzhou Affiliated to University of South China, Chenzhou 423000, P. R. China.

[5]Research Institute, DAAN Gene Co., Ltd., Guangzhou 510665, P. R. China.

*Corresponding authors E-mail: luodixian_2@163.com; liutc@smu.edu.cn; liu_quan2020@163.com

Supplementary Methods

Site-directed mutagenesis and Sanger sequencing

Primers containing mutated bases were used to amplify the templates of *EGFR* T790M and C797S wild type or mutant type. The PCR reaction contained 1×Taq mix (Vazyme, Cat# P111), 0.3 μM primers, and 1 ng template, and the procedure was carried out as follows: 95°C for 1 min; 35 cycles of 95°C for 20 s, 55°C for 15 s, and 72°C for 30 s; and holding at 72°C for 5 min. The PCR products were sent for Sanger sequencing (Ruibio Biotech, Guangzhou, China). The sequencing primer used was 5'-CAGATGACTGTAATACGA-3' and the sequencing map was analyzed with Chromas Software version 2.6.6. For determining the mutated positions of T790M and C797S, the 1% VAF T790M-cis-C797S was mixed with 10^{-3} ng of wild-type plasmid, 10^{-5} ng of C797S mutated plasmid, and 10^{-5} ng of T790M mutated plasmid. The 1% VAF T790M-trans-C797S was mixed with 10^{-3} ng of wild-type plasmid and 10^{-5} ng of T790M/C797S double-mutated plasmid. About 100 ng of PCR products were digested by the HpyCh4V (NEB, Cat# R0620S), enzyme at 37°C for 40 min. The digested products were purified using the Select-a-Size DNA Clean & Concentrator Kit (Zymo Research, Orange County, China, Cat# D4080) following the manual. The volumes of 140 and 290 μL of 95% ethanol (Sigma, Cat# 493511) were employed to isolate the digested products into different sizes. The purified products were detected with Cas13a/crRNA reaction for 40 min using Real-Time PCR Systems.

DNA cleavage assay of restriction enzyme

The primers of F1/R1 were used to amplify plasmids of 19 dels and wild type as the DNA templates for MseI (NEB, Cat# R0525S) digestion. For L858R, PCR products amplified with

primers F1/R1 were used to be digested by the MscI (NEB, Cat# R0534S) enzyme. PCR products of T790M after site-directed mutagenesis were subjected to digesting by the SsiI (Thermo Fisher Scientific, Cat# ER1791) enzyme, and the T790M/C797S mutations were digested by the HpyCh4V enzyme. The above reaction system of 20 μL mainly included 1× Cutsmart buffer, 40 ng DNA templates, 5 U RE, and DEPC H_2O, and was incubated at 37°C for 40 min. The digested products were delivered to 2% agarose gel and then observed on a gel imager system.

Detection of *EGFR* mutations by Super-ARMS assay

EGFR dels, L858R, and T790M mutations were detected by the Super-ARMS assay. The human *EGFR* Gene Mutation Quantitative Detection Kit was purchased from AmoyDx (Xiamen, China). A total of 2 μL cfDNA was added into the mixture for amplification using Real-Time PCR Systems by the manufacturer's instruction. Internal control was employed to monitor the quality of the sample, and evaluating the CT difference between the target products and the internal control determined the presence of mutations in this sample.

Supplementary Figures

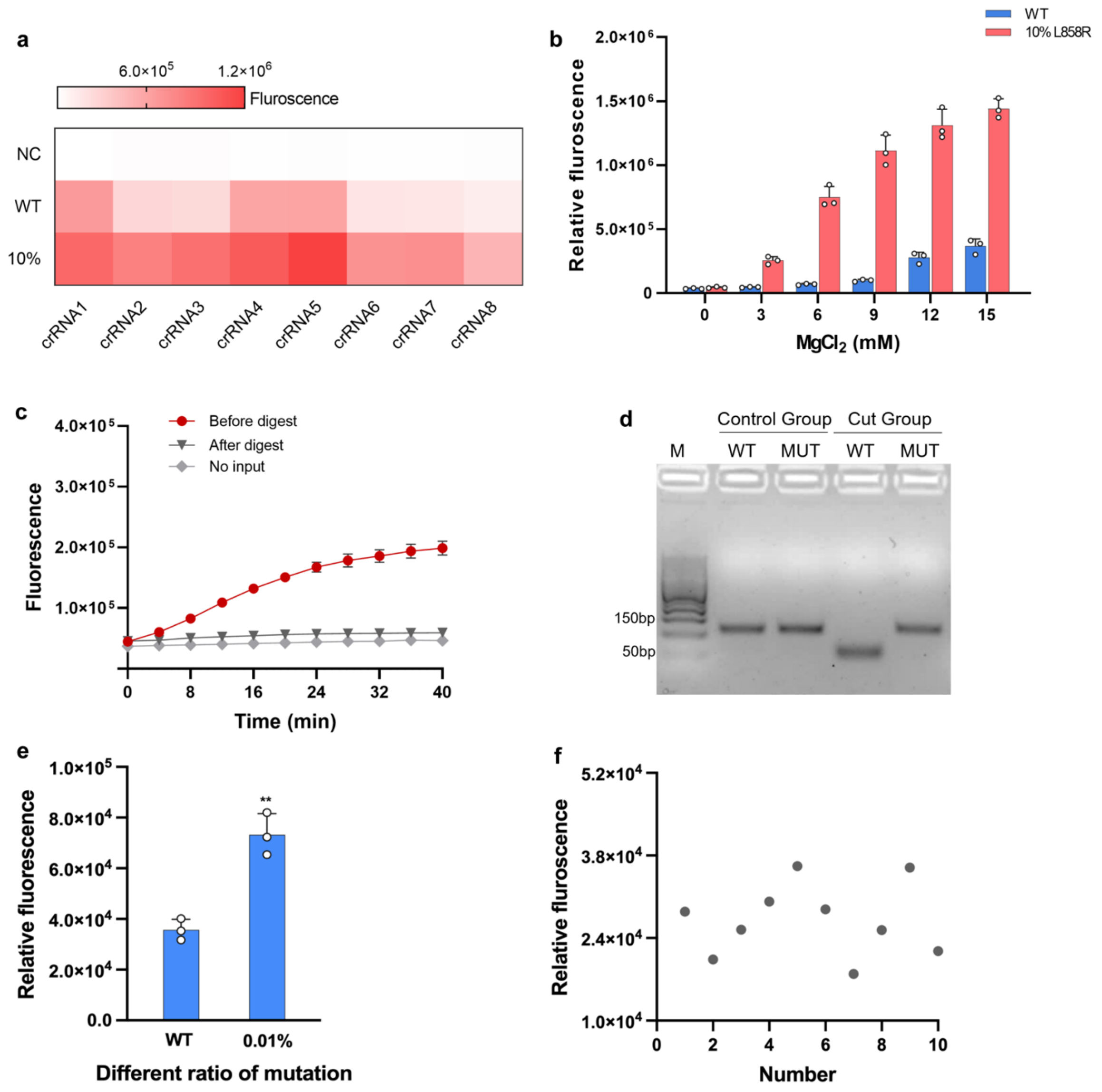

Supplementary Figure 1. The screening of crRNA and optimization enzyme digestion condition of L858R nutation. a Screening out optimal crRNA targeting L858R from eight crRNAs. 10% was mixed by wild-type and mutant-type templates. **b** Screening out optimal concentration of $MgCl_2$ with 3 mM, 6mM, 9mM, 12mM, 15mM. **c** Real-time fluorescence detection for L858R wild-type fragments before and after digestion by MscI enzyme. **d** The PCR products before and after digestion by MscI enzyme were analyzed on this agarose gel. WT, wide type; MUT, mutant type; M, 50 bp marker of DNA. **e** Detection of the VAF of 0.01% in L858R with the input of 40 ng cfDNA standard. **f** The detection limit of *EGFR* T790M. n =3

independent experiments; The error bars indicate standard deviation.

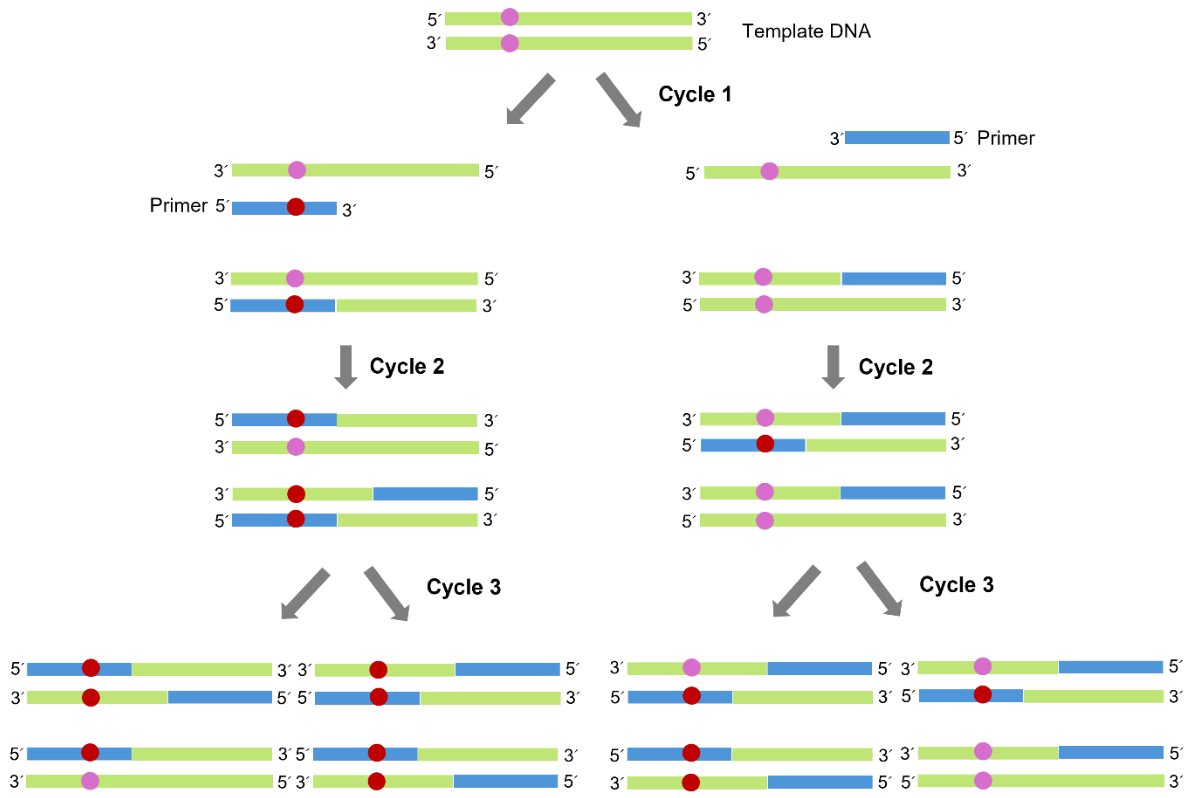

Supplementary Figure 2. Schematic diagram of PCR site-directed mutagenesis. The purple point indicates the original base, and the red point indicates the mutated base. The primer carrying the mutation is employed to replace the original base via PCR amplification. Since Taq DNA polymerase lacks 3' to 5' exonuclease activity, it cannot identify a mismatch between the primer and the template. After the initial cycle, the product yielded ssDNA containing the mutated base. After the second cycle, the product generated dsDNA with the mutated base. By the third cycle, it consisted of 50% dsDNA with the mutated base, and after four cycles of amplification, nearly all the product contained mutated dsDNA.

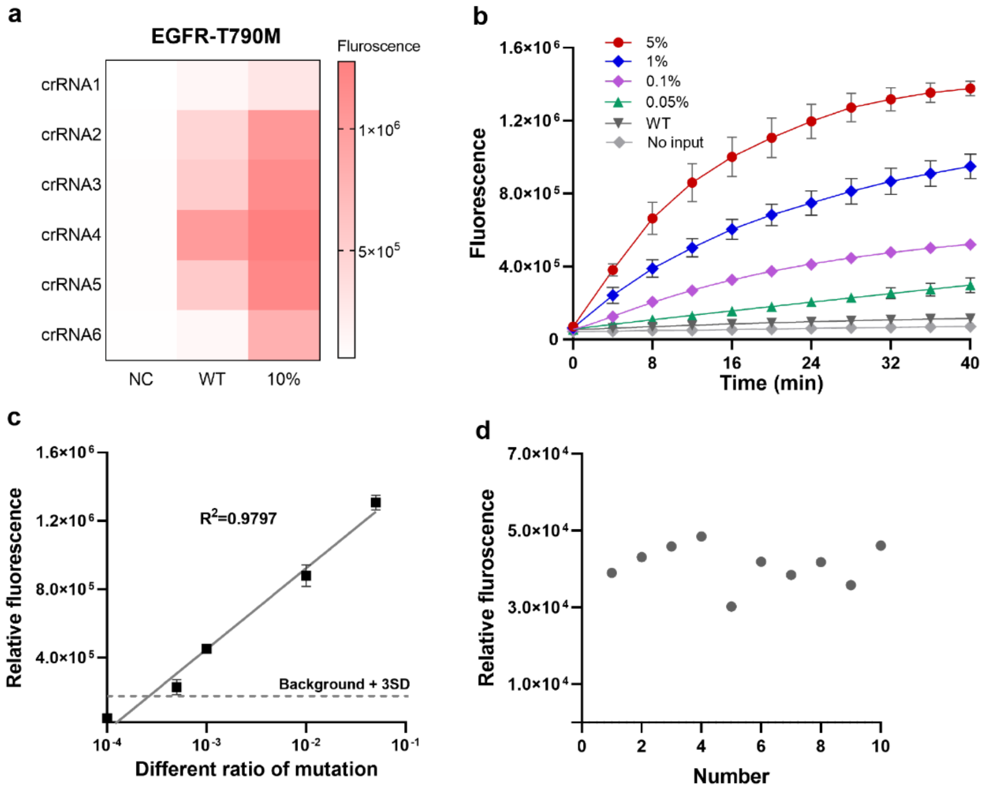

Supplementary Figure 3. Establishing standard detection system of T790M using the HiCASE assay.

a Screening out optimal crRNA targeting T790M from six crRNAs. **b** Real-time fluorescence detection of T790M with different VAFs of cfDNA standards using the HiCASE assay. **c** The standard curve of T790M indicated the correlation of fluorescent value with different VAFs by the HiCASE assay. **d** The detection limit of *EGFR* T790M. The error bars indicate standard deviation. *n* =3 independent experiments.

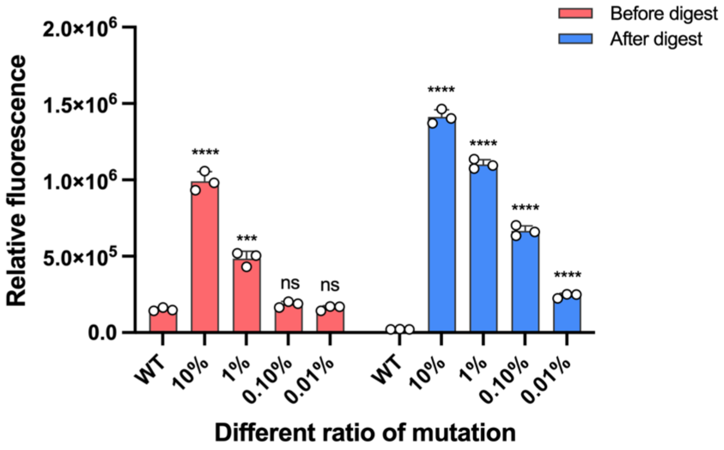

Supplementary Figure 4. The detection of different VAFs of *EGFR* 19del plasmids using two approaches. The data was analyzed using two-tailed Student's t test. $n = 3$ independent experiments. The error bars indicate standard deviation; ***P < 0.001, and ****P < 0.0001; ns, not significant.

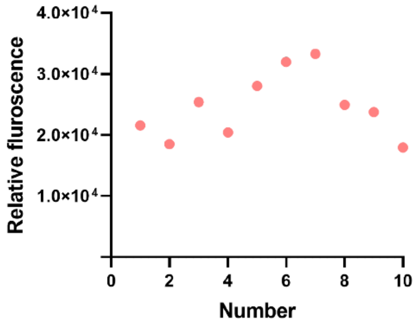

Supplementary Figure 5. The detection limit of *EGFR* 19dels.

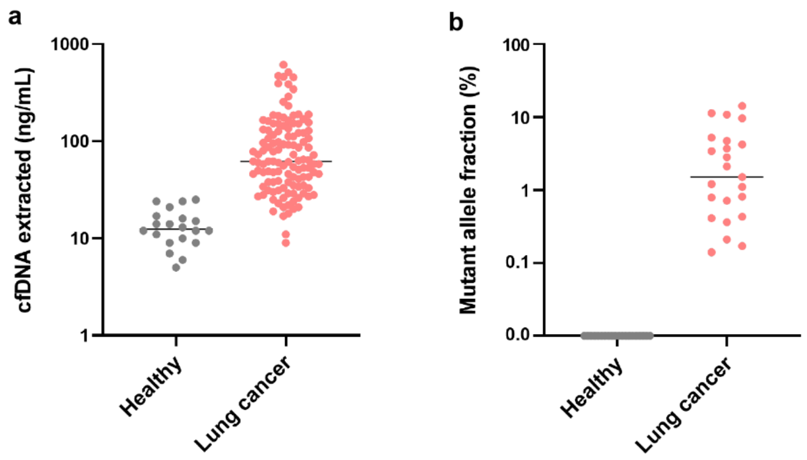

Supplementary Figure 6. cfDNA in healthy individuals and patients with lung cancer. a The concentration of cfDNA in the plasma of healthy individuals and lung cancer patients. **b** The mutant allele fraction of EGFR detected by ddPCR in healthy individuals and lung cancer patients. Medians for each group are represented by the black bars.

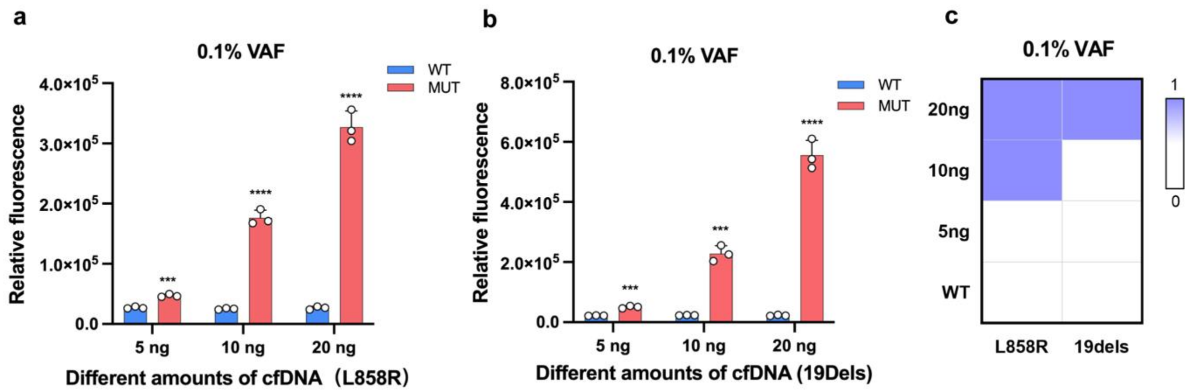

Supplementary Figure 7. Comparation of different amounts of cfDNA used by the HiCASE and ddPCR assays. a-b The results of detecting L858R and 19dels mutations using the HiCASE assay with different amounts of cfDNA standards in 0.1% VAFs. **c** The detection of L858R and 19dels used by ddPCR in different amounts of cfDNA standards with 0.1% VAFs. The error bars indicate standard deviation. *n* =3 independent experiments.

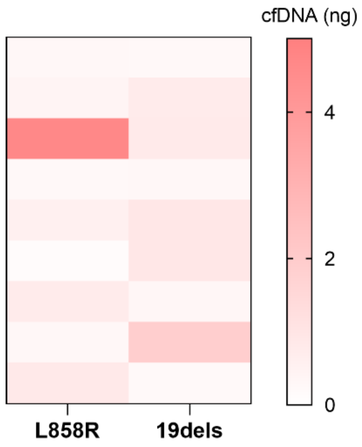

Supplementary Figure 8. The minimum amount of cfDNA for detecting L858R and 19dels mutation calculated from Figure 5a.

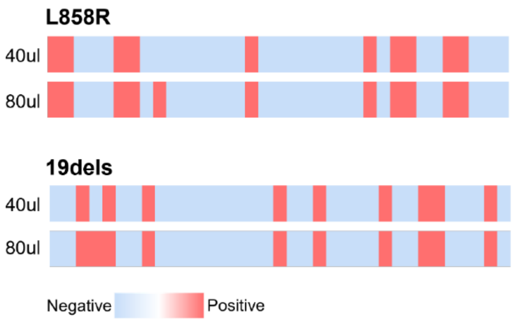

Supplementary Figure 9. Comparison of evaluating the performance of the Super-ARMS assay in identifying L858R and 19dels mutations with 40ul and 80ul plasma.

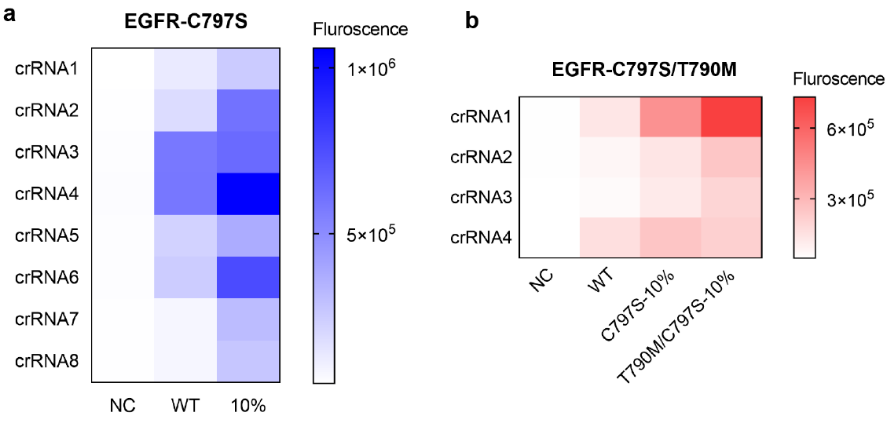

Supplementary Figure 10. The screening of crRNA of T790M/C797S mutations. a Screening out optimal crRNAs targeting C797S from eight crRNAs. **b** screening out optimal crRNA for T790M-cis-C797S mutations with shorter spacer sequence.
