# Supplementary material for: CRISPR/Cas13a-based supersensitive circulating tumor DNA assay for detecting EGFR mutations in plasma
Source: Commun Biol. 2024 May 28;7:657. doi: 10.1038/s42003-024-06368-2 (PMC11133305; doi:10.1038/s42003-024-06368-2)
Supplement: Supplementary file 3 — Description of Additional Supplementary Files [file 42003_2024_6368_MOESM3_ESM.pdf]

Description of Additional Supplementary Files

File: Supplementary Data 1.

Description: Detecting blood samples of NSCLC patients with HiCASE, ddPCR and Super-ARMS assays

File: Supplementary Data 2.

Description: Clinical characteristics of HiCASE assay compared to ddPCR and Super-ARMS assays in detecting EGFR mutations.

File: Supplementary Data 3

Description: The detected resluts of L858R and 19dels mutations with different volume of plasma using HiCASE and dPCR assays.

File: Supplementary Data 4

Description: Different volumes of plasma used by Super-ARMS assay.

File: Supplementary Data 5

Description: The sequence of crRNA and primers.

File: Supplementary Data 6

Description: All source data used for generating graphs and charts in main figures.
