# Supplementary figures and images for: CRISPR/Cas13a-based supersensitive circulating tumor DNA assay for detecting EGFR mutations in plasma
Source: Commun Biol. 2024 May 28;7:657. doi: 10.1038/s42003-024-06368-2 (PMC11133305; doi:10.1038/s42003-024-06368-2)

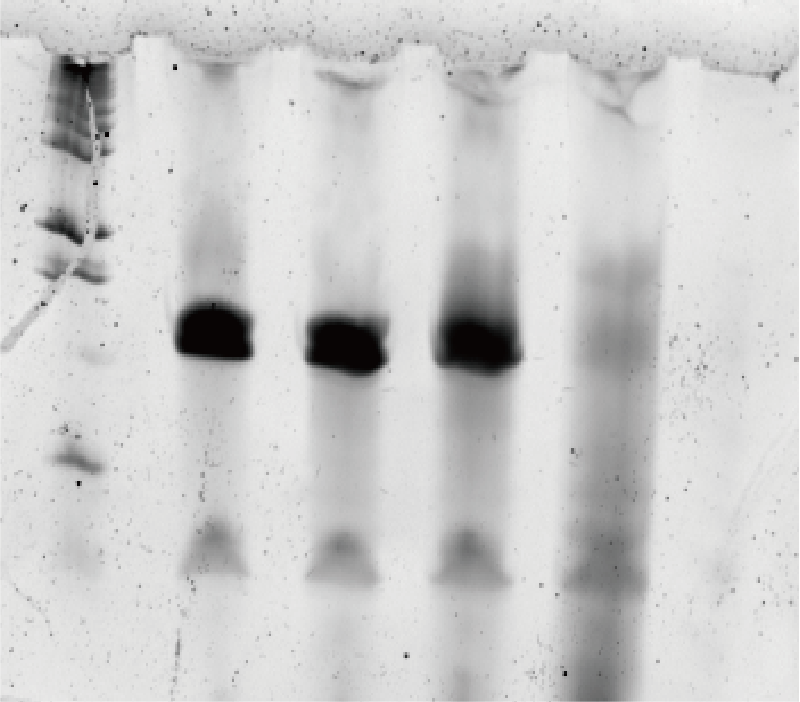

Supplement: Supplementary file 5 — Supplementary Data 6 [file 42003_2024_6368_MOESM5_ESM.zip › Supplementary Data 6/Unedited gels/Figure 2a.tif]

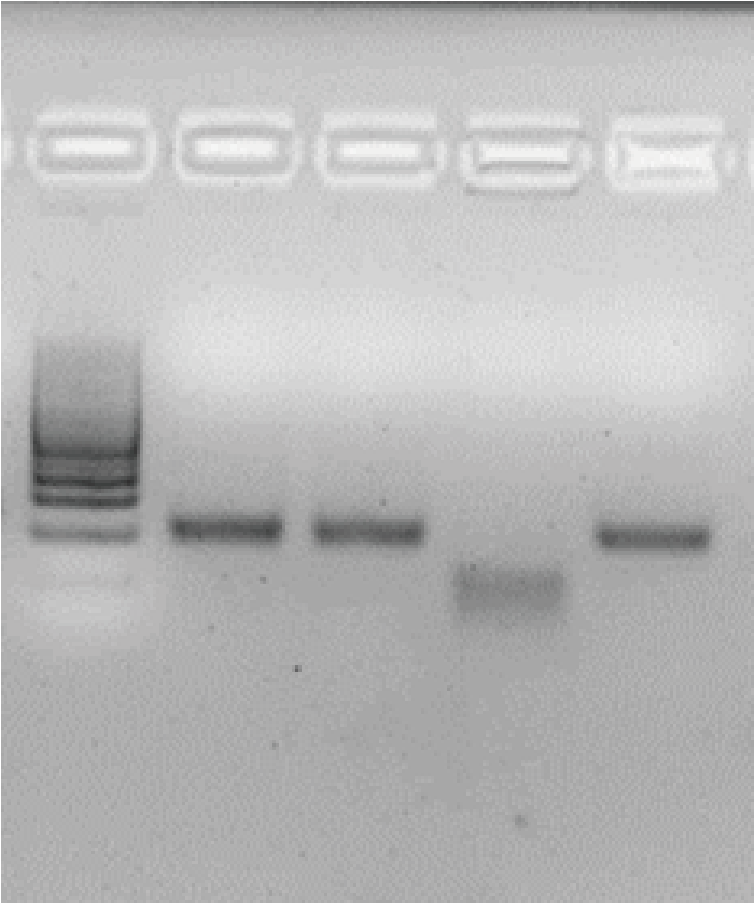

Supplement: Supplementary file 5 — Supplementary Data 6 [file 42003_2024_6368_MOESM5_ESM.zip › Supplementary Data 6/Unedited gels/Figure 2g.tif]

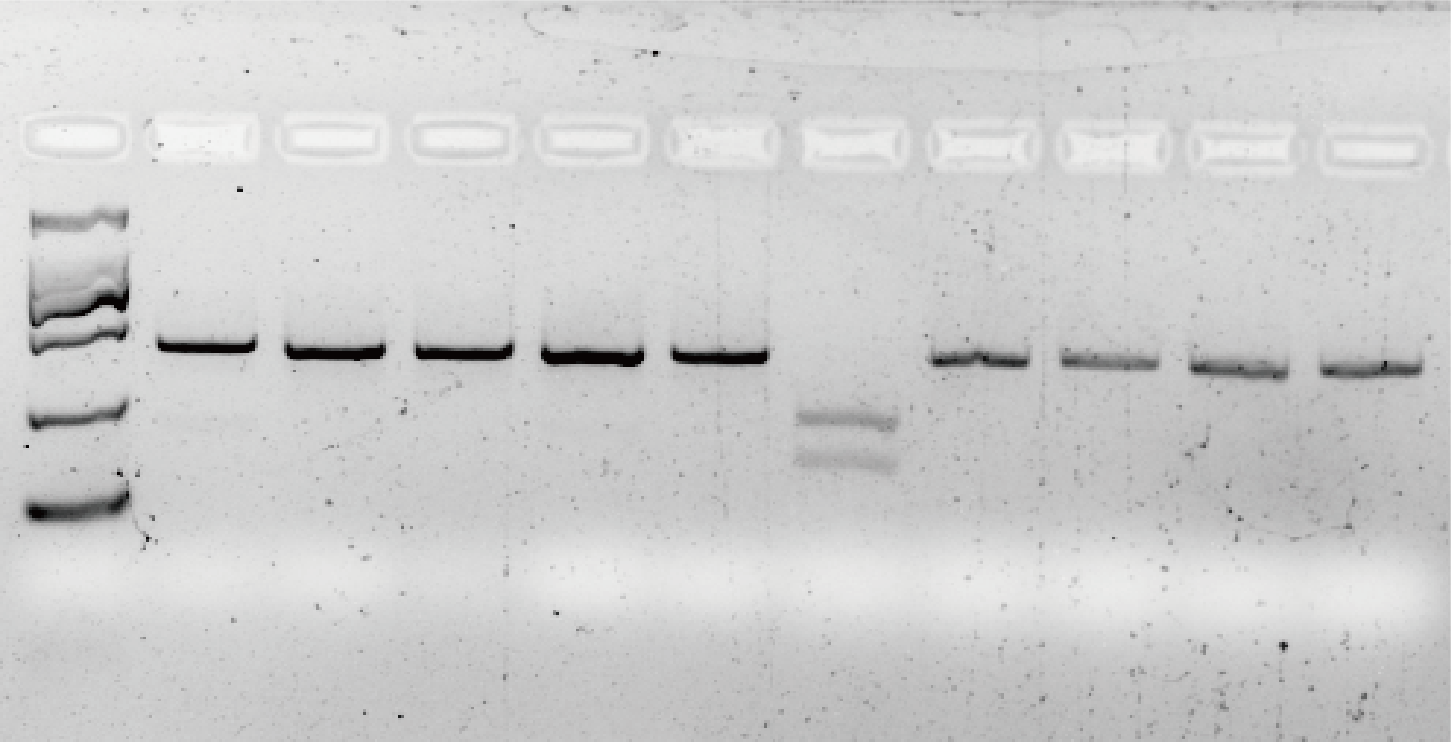

Supplement: Supplementary file 5 — Supplementary Data 6 [file 42003_2024_6368_MOESM5_ESM.zip › Supplementary Data 6/Unedited gels/Figure 3c.tif]

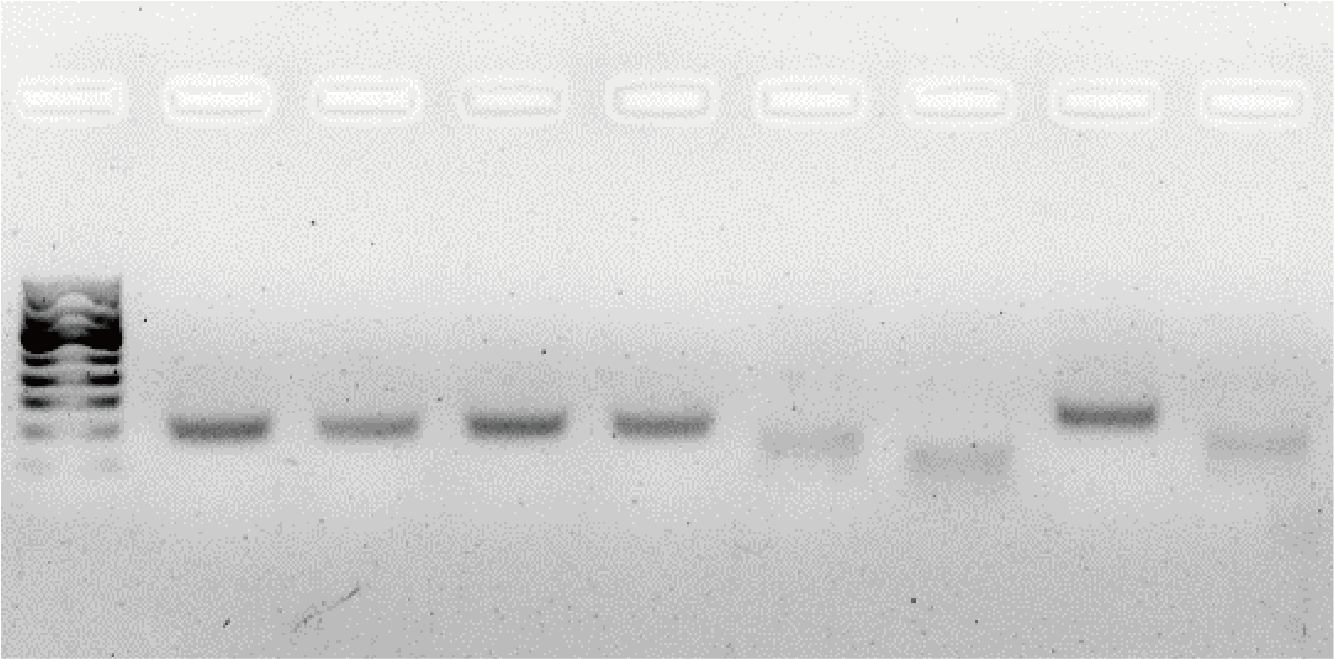

Supplement: Supplementary file 5 — Supplementary Data 6 [file 42003_2024_6368_MOESM5_ESM.zip › Supplementary Data 6/Unedited gels/Figure 6b.tif]

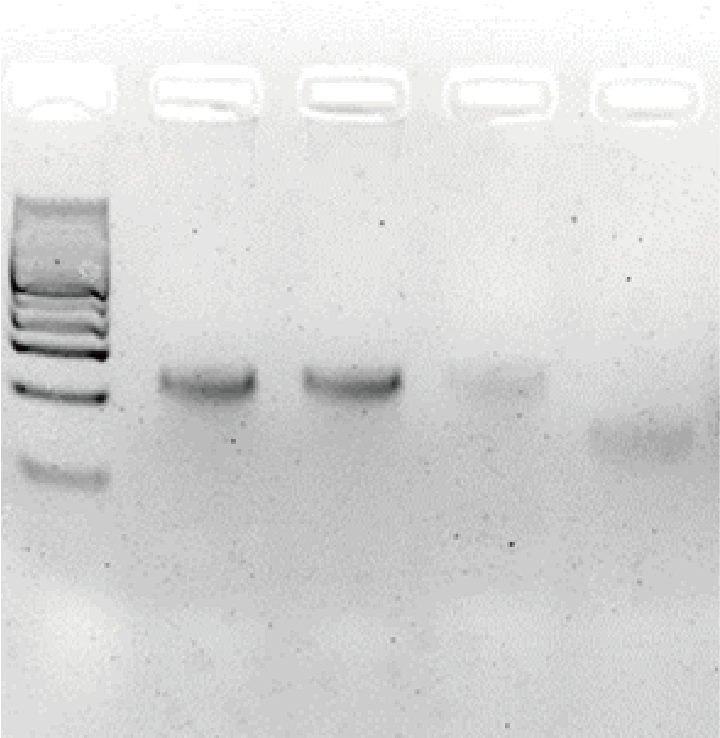

Supplement: Supplementary file 5 — Supplementary Data 6 [file 42003_2024_6368_MOESM5_ESM.zip › Supplementary Data 6/Unedited gels/Figure 6c.tif]

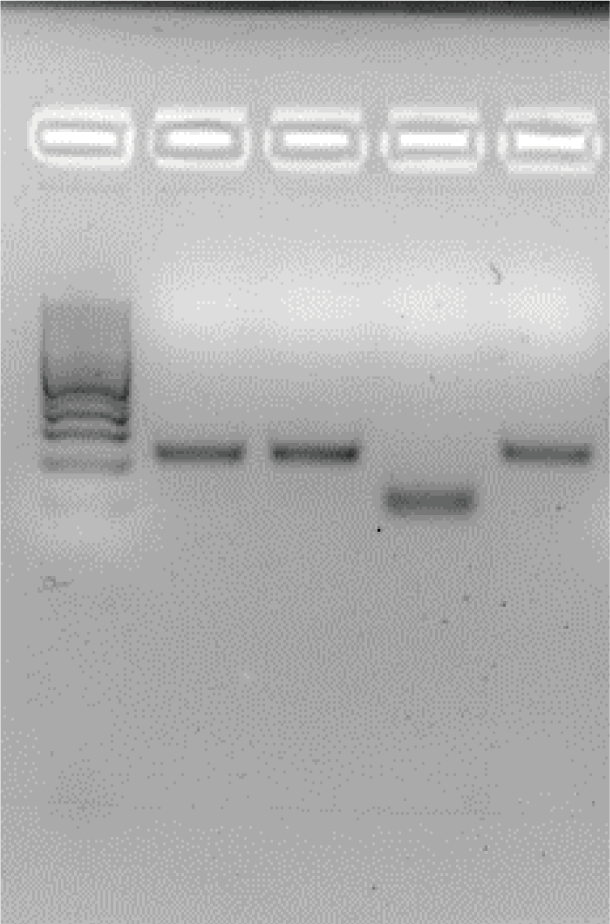

Supplement: Supplementary file 5 — Supplementary Data 6 [file 42003_2024_6368_MOESM5_ESM.zip › Supplementary Data 6/Unedited gels/Supplementary figure 1d.tif]
